# Comparative Transcriptional Analysis of Loquat Fruit Identifies Major Signal Networks Involved in Fruit Development and Ripening Process

**DOI:** 10.3390/ijms17111837

**Published:** 2016-11-04

**Authors:** Huwei Song, Xiangxiang Zhao, Weicheng Hu, Xinfeng Wang, Ting Shen, Liming Yang

**Affiliations:** 1 Jiangsu Key Laboratory for Eco-Agricultural Biotechnology around Hongze Lake, College of Life Science, Huaiyin Normal University, Huai’an 223300, Jiangsu, China; xxzhao2013@163.com (X.Z.); hu_weicheng@163.com (W.H.); wangxf@hytc.edu.cn (X.W.); shenting1019@163.com (T.S.); yanglm@hytc.edu.cn (L.Y.); 2 Jiangsu Collaborative Innovation Center of Regional Modern Agriculture & Environmental Protection, Huaiyin Normal University, Huai’an 223300, Jiangsu, China

**Keywords:** *Eriobotrya japonica*, transcriptome analysis, fruit development, ripening-regulated genes, expression profile

## Abstract

Loquat (*Eriobotrya japonica* Lindl.) is an important non-climacteric fruit and rich in essential nutrients such as minerals and carotenoids. During fruit development and ripening, thousands of the differentially expressed genes (DEGs) from various metabolic pathways cause a series of physiological and biochemical changes. To better understand the underlying mechanism of fruit development, the Solexa/Illumina RNA-seq high-throughput sequencing was used to evaluate the global changes of gene transcription levels. More than 51,610,234 high quality reads from ten runs of fruit development were sequenced and assembled into 48,838 unigenes. Among 3256 DEGs, 2304 unigenes could be annotated to the Gene Ontology database. These DEGs were distributed into 119 pathways described in the Kyoto Encyclopedia of Genes and Genomes (KEGG) database. A large number of DEGs were involved in carbohydrate metabolism, hormone signaling, and cell-wall degradation. The real-time reverse transcription (qRT)-PCR analyses revealed that several genes related to cell expansion, auxin signaling and ethylene response were differentially expressed during fruit development. Other members of transcription factor families were also identified. There were 952 DEGs considered as novel genes with no annotation in any databases. These unigenes will serve as an invaluable genetic resource for loquat molecular breeding and postharvest storage.

## 1. Introduction

Loquat (*Eriobotrya japonica* Lindl.) is a subtropical evergreen fruit tree that blooms in fall and early winter. It is commercially grown in many countries, including China, Japan, Brazil, and Spain [1]. Loquat fruit is popular for its medical value, juicy taste, and high content of phytochemicals such as carotenoids and flavonoids [2]. These beneficial properties are especially influenced by gene types and their different expressions during fruit development and ripening. Although the molecular mechanism has been extensively studied in climacteric fruits like tomato [3,4,5,6], it has not been well explored in non-climacteric fruits until now. Loquat fruit, a non-climacteric fruit, undergoes rapid changes in sugars, pigments, organic acids, and other components during fruit development. Wide changes of biochemical and physiological characteristics were also found among different loquat cultivars [7,8]. Therefore, loquat fruit provides a very useful model for studying the molecular mechanism related to fruit component alteration and nutrient metabolism during non-climacteric fruit development.

Despite the nutritional and economic importance of loquat fruit, there is no genomic resource for this non-model species. Current biological studies mainly focus on genetic map construction, post-harvest physiology, and fruit preservation [9,10,11,12]. Very little research based on genetic and molecular levels has been carried out on the species due to its complex genetic background, large genome size, poor transgenic system, and long life cycle. To date, several genes involved in ethylene biosynthesis and lignin metabolism have been identified [13,14,15,16], suggesting a potential function during fruit development and softening. However, an integrity transcriptome of loquat fruit remains unavailable.

Currently, the cDNA library and RNA-Seq are widely applied for rapid gene discovery, especially in non-model organisms. The SMART^TM^ technology for high quality cDNA library construction is very straightforward and robust [17]. For woody plants, especially those of high heterozygosity like loquat, whole genome sequencing requires a long-term and expensive investment. Instead, it has been more useful to obtain unigene information through RNA-Seq. Transcriptome analysis provides an insight into functional genes and signal pathways involved in fruit ripening without the corresponding genome information as a reference [18,19,20,21,22]. These data were used to investigate allele-specific expression and splice junction variation. Furthermore, a rich source of gene-derived molecular markers can be also detected, and widely used for germplasm breeding or physical map construction [19].

Here, RNA samples of loquat fruit (cv. Jiefangzhong) from three different stages were sequenced using the latest Illumina deep sequencing technique. The data provided an overview that the transcription characteristics and signal networks of candidate genes were involved in regulating fruit development and ripening. Some differentially expressed genes (DEGs) were uncovered such as, sugar metabolism genes, auxin metabolism regulators, ethylene-responsive elements, and expansin genes (*EjEXPAs*). The majority of transcription factors (TFs) were also identified. Real-time reverse transcription (qRT)-PCR analysis revealed that their expression levels demonstrated significant alterations during fruit development. Therefore, the Solexa/Illumina RNA-seq high-throughput sequencing offers a helpful tool for exploring some valuable and rare genes to assist fruit breeding.

## 2. Results

### 2.1. Changes in Sugar Components, Total Soluble Solids (TSS) and Titratable Acidity (TA)

**Figure 1:**
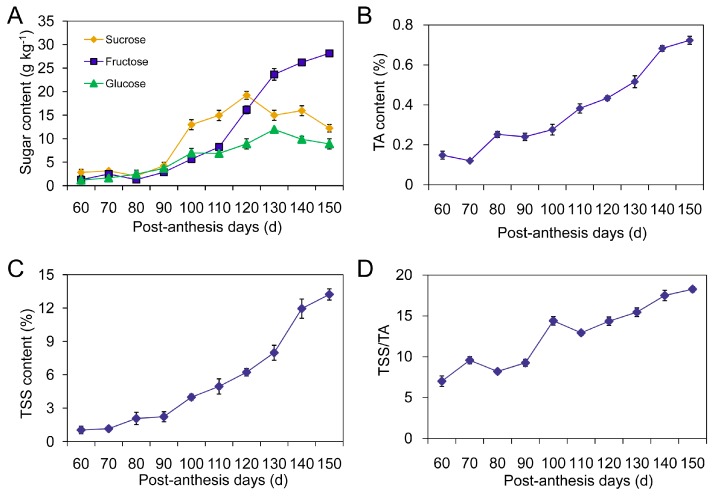
Changes in sugar components (**A**); titratable acidity (TA) (**B**); total soluble solids (TSS) (**C**) and TSS/TA (**D**) during development and ripening of loquat fruit. The presented data are the mean of three replicates (25 fruit samples each) from three experiments. Vertical bars, if larger than symbols, represent the standard errors of the mean (*n* = 3).

The quality of loquat fruit is closely related to sugar accumulation and Total Soluble Solids/Titratable Acidity (TSS/TA) ratio. We found that fructose, glucose, and sucrose were three major sugars detected in cv. Jiefangzhong. During the initial stage of fruit development, total sugar content was 7.3 g·kg^−1^. After 90 post-anthesis days (PAD), sucrose accumulation was faster than fructose and glucose accumulation, reaching a peak value on day 120, and then declining by the end of fruit ripening (Figure 1A). By contrast, fructose dramatically increased after 110 PAD and was a predominant component in ripe loquat fruit, accounting for 57.1% of total sugars. Although glucose also increased during fruit development, its percentage relative to total sugars decreased from 22.4% to 18.1%.

TA gradually increased during fruit development and reached the highest concentration of 0.7% on day 150 (Figure 1B). Although no obvious change of TSS content was observed during the first 10-day interval (60–70 PAD), the continuous increase was demonstrated from 60 to 150 PAD, (Figure 1C). Especially at the stage of fruit ripening, TSS was significantly accumulated and reached a peak value (13.2%) until harvest. By contrast, the TSS/TA ratio showed a constant increment from 9.6% to 18.3%, and exhibited two peaks on days 70 and 100, respectively (Figure 1D). Based on the dynamic changes of physiological indexes, loquat fruit development can be divided into three stages: cell division (stage I marked from 60 to 90 PAD), an expansion phase involving cell enlargement and sugar accumulation (stage II marked from 90 to 120 PAD), and ripening (stage III marked from 120 to 150 PAD).

### 2.2. Generation of a Full-Length Enriched and Normalized cDNA Library

**Figure 2:**
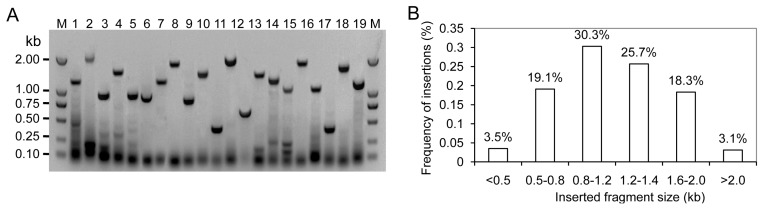
Length and fullness ratios of inserts evaluated by PCR amplification. (**A**) The partial results detected by PCR amplification. 1–19: Electrophoresis results of PCR production; M: Marker DL 2000; (**B**) The distribution frequency of inserts.

To obtain a global overview of the transcriptome during fruit development, an equal amount of total RNAs from each of three stages was pooled for RNA-Seq. The SMART™ RNA oligo was added to the reaction buffer for the enriched long cDNAs. The double-strand fraction formed by abundant transcripts was removed using duplex-specific nuclease (DSN). Following SMART™ cDNA synthesis, the selective PCR amplification was performed to enhance the abundance of rare genes. More PCR cycles were carried out on the larger fractions to obtain a normalization amount. The amplified cDNAs were used for cDNA library construction. There were 375 clones randomly selected to investigate the length and fullness ratios of insertions by PCR amplification (Figure 2A). The insert size ranged from 0.6 to 3.5 kb with an average insert size of 1.1 kb (Figure 2B). The primary titer of cDNA library was 1 × 10^6^ cfu·mL^−1^.

### 2.3. Transcriptomic Analysis of Loquat Fruit

Over 2.04 G of 56 nt single-end read data were produced with a Q20 percentage (sequencing error rate < 1%) of 96.5%. The percentage of unassigned base “N” was 0.00% and the average GC content was 43.5%. The high quality reads were assembled into 48,838 unigenes with a mean size of 790 bp (Table 1). By contrast, the redundancy ratio was sharply decreased in a clustering analysis of all sequences generated from the normalized library using the program Megalign (Lacergene, DNAstar, Inc., Madison, WI, USA). The ratio of non-redundant unigenes was 70.3%.

### 2.4. Sequence Annotation

**Figure 3:**
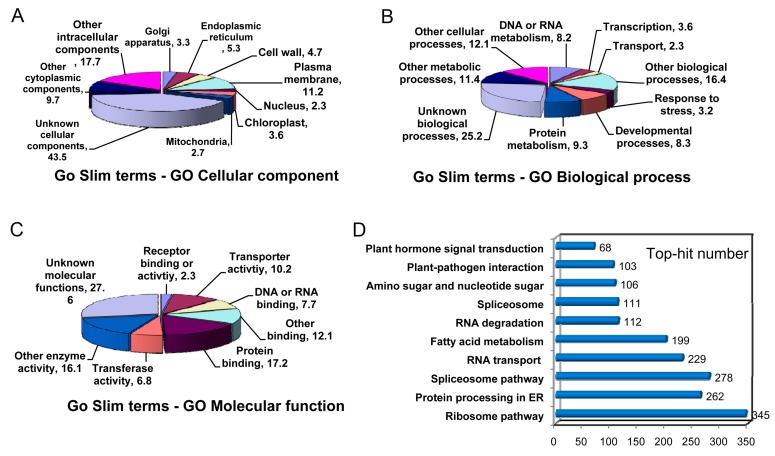
Functional categorizations of unigenes predicted by Gene Ontology (GO) terms and Kyoto Encyclopedia of Genes and Genomes (KEGG) pathway. Many unigenes were distributed into the cellular component (**A**); molecular function (**B**); and biological process (**C**) categories. Top 8–10 GO terms were demonstrated in each category; (**D**) The unigene number was shown in the top 10 KEGG pathways.

The Gene Ontology (GO) annotation was performed with the Blast2GO software [23]. Out of 48,838 unigenes, 31,034 were classified into the “cellular component”, “biological process”, and “molecular function” categories (Figure 3). This classification provided some information on the percentage of loquat unigenes involved in different signal transductions, catabolic and anabolic processes. For the cellular component category, the majority of unigenes was grouped into “other intra-cellular components,” “unknown cellular components” and “other cytoplasmic components,” which accounted for about 70.9% of all annotated unigenes (Figure 3A). Although non-green tissue was used for cDNA library construction, 3.6% of unigenes belonged to “chloroplast cellular component”. In the biological process category, the unigenes were mainly distributed into nine different metabolism processes such as “protein metabolism” (9.3%), “developmental process” (8.3%), “response to stress” (3.2%), “transcription” (3.6%) and “an unknown item”. The larger unigenes were involved in “other metabolic process” (11.4%), “other cellular process” (12.1%), “other biological process” (16.4%) and “unknown biological process” (25.2%) (Figure 3B). For the molecular function category, a large number of unigenes was distributed into “transferase activity” (6.8%), “transporter activity” (10.2%) and “binding activities” (24.9%) (Figure 3C). Greater proportions of unigenes were classified under “unknown molecular function”, “other enzyme activity” and “other binding”, which accounted for 55.8% of all unigenes.

In addition, 29,987 out of 48,838 unigenes were mapped into 120 Kyoto Encyclopedia of Genes and Genomes (KEGG) pathways. The map with the abundant unigenes is demonstrated in Figure 3D, including 345 unigenes in “ribosome pathway (ko03010)”, 278 unigenes in “spliceosome pathway (ko03040)”, 262 unigenes in “protein processing in endoplasmic reticulum (ko04141)” and 229 unigenes in “RNA transport (ko03013)”. Notably, 68 unigenes were observed in “plant hormone signal transduction (ko04075)”.

### 2.5. Differentially Expressed Genes (DEGs) Involved in Fruit Development and Ripening

**Figure 4:**
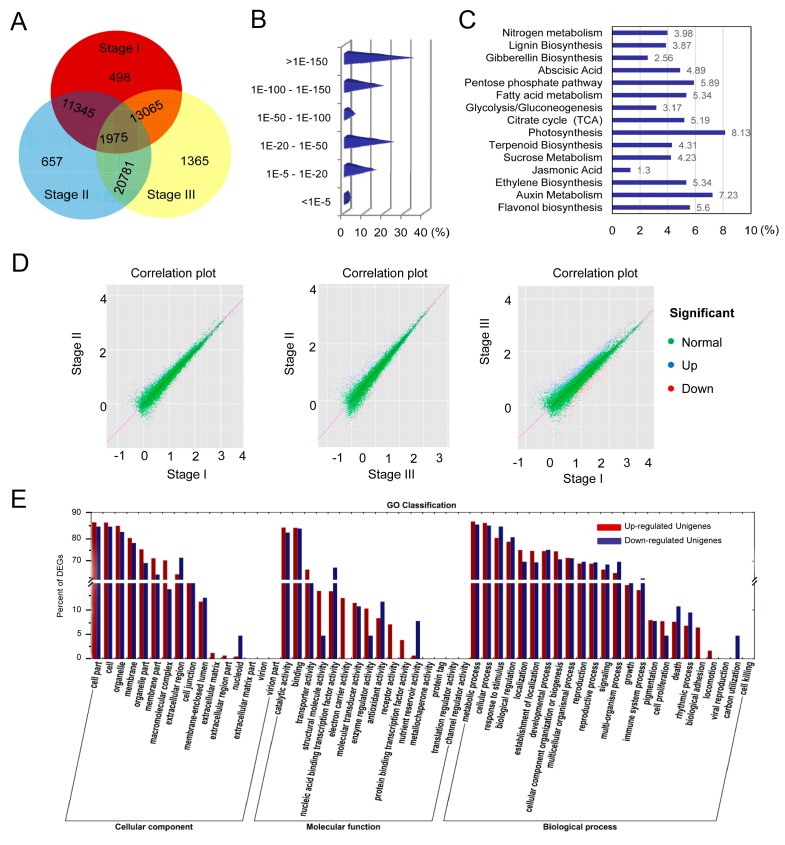
Homology alignments and expression analyses of DEGs. (**A**) Venn diagram of differentially expressed genes (DEGs) annotated by BlastX with an *E-*value threshold of 10^−5^ against protein databases; (**B**) *E*-value distribution of DEGs against nr database; (**C**) Overall 15-top categories involved in the signal pathways of biosynthesis and metabolism; (**D**) Distributions of correlation plots and expression characteristics of unigenes between both of stages; (**E**) GO classification of upregulated and downregulated unigenes.

To identify differentially expressed genes (DEGs) in each stage of fruit development, the distribution of unique reads was calculated (Figure 4A). Similar patterns throughout the abundance categories were observed among the three libraries (Table S1). Moreover, unigene transcription profiles were analyzed using the uniquely mapped DEG reads. Results from two biological replicates were highly similar, suggesting a good reproducibility of the method. A total of 3256 unigenes referred to as DEGs were used for the subsequent analysis. Subsequently, 1987 unigenes could be annotated to non-redundant (nr) database and 2304 unigenes could be annotated to all databases. Based on nr annotation and *E*-value distribution, 17.0% of the mapped unigenes showed a strong homology (*E*-value < 20) with available plant sequences (Figure 4B). The 30 top hit DEGs against nr annotation are shown in Table 2.

The enrichment analysis in the KEGG pathway revealed that 41.7% (1358/3256) of DEGs were involved in 119 KEGG pathways, indicating the complete coverage of the transcriptome (Table S2). The majority of DEGs was mapped into “metabolism”, “genetic information processing”, “cellular process”, “environmental information”, and “organism systems”. The abundant metabolism pathways, including carbohydrate, fatty acid, energy, lignin and hormone metabolism, are demonstrated in Figure 4C. According to the distribution of correlation plots between both stages, many DEGs were mainly scattered between stage I and stage III (Figure 4D).

GO-term analysis presented that DEGs within the upregulated and downregulated cluster groups were classified into “cellular component”, “molecular function” and “biological process”. Under the cellular component category, a great number of DEGs were mainly categorized as “cell part”, “cell”, and “organelle” (Figure 4E). For the molecular function category, catalytic and binding activities were considered as the top abundant subcategories. Under the biological process category, most of those were classified into “cellular process”, “metabolic process”, and “response to stimulus”. More than 125 DEGs were associated with “carbohydrate”, “primary and secondary metabolism” as well as “lipid metabolic process”, reflecting the dynamic alteration processes during fruit development.

### 2.6. DEGs Enriched in the Sugar-Metabolism Signalling Pathway

**Figure 5:**
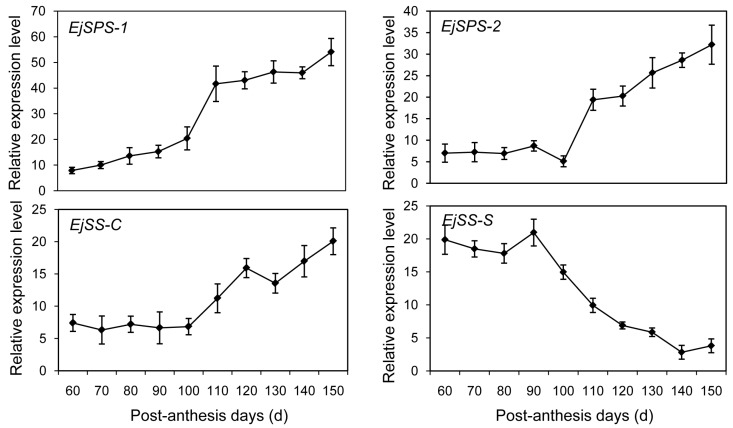
Expression profiles of loquat sucrose synthase and sucrose phosphate synthase (*EjSPS* and *EjSS*) using qRT-PCR during fruit development. Each point is the mean of three determinations. Vertical bars, if larger than symbols, represent the standard error of the mean (*n* = 3).

Soluble sugar accumulation underwent a dynamic change during loquat fruit development (Figure 1). A comprehensive understanding of the sugar accumulation cycle will improve fruit quality. Currently, attempts to elucidate sucrose metabolism mainly focus on sugar metabolism enzymes like sucrose synthase (SS) and sucrose phosphate synthase (SPS). SS catalyzes a reversible reaction of sucrose synthesis and hydrolysis in the synthetic (SS-S) and cleavage (SS-C) directions, respectively. Until now, the genes encoding SS and SPS in loquat (EjSS and EjSPS) have not been reported. In the present study, homology genes such as *EjSPS1*, *EjSPS2*, *EjSS-C* and *EjSS-S* were identified based on the transcriptome data. qRT-PCR was used to evaluate their expression profiles. The expression levels of *EjSPS1* gradually increased during the whole development stages of loquat fruit. A sharp rise was detected on day 110 and there was no significant expression difference observed from 110 to 150 PAD. In contrast to low mRNA level from 60 to 90 PAD, the transcription levels of *EjSPS2* dramatically increased after 100 PAD (Figure 5). A similar change pattern was exhibited in the expression characteristics of *EjSS-C*. However, *EjSS-S* showed a significant decrease from 90 to 150 PAD, and no differential expression was observed from 60 to 90 PAD.

### 2.7. DEGs Significantly Enriched in the Hormone-Mediated Signaling Pathways

**Figure 6:**
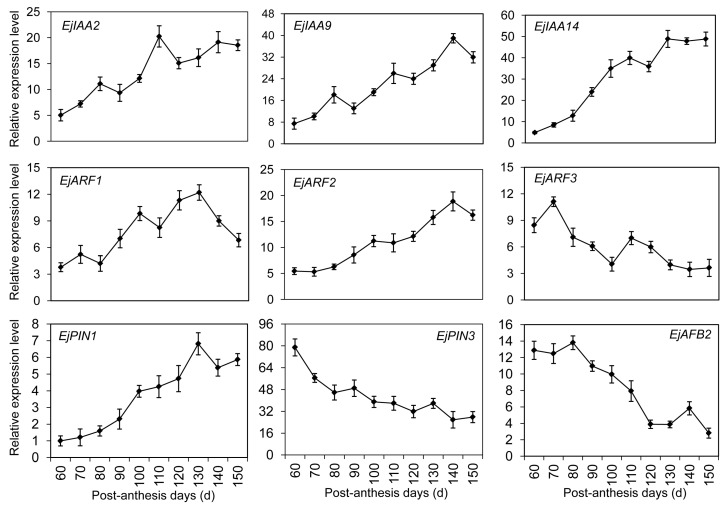
Expression profiles of differentially expressed genes DEGs involved in the auxin signal pathway using qRT-PCR. Each point is the mean of three determinations. Vertical bars, if larger than symbols, represent the standard error of the mean (*n* = 3).

A total of 72 DEGs were mapped into different hormone pathways against the *Arabidopsis* hormone database, especially in auxin and ethylene signaling pathways. They were associated with hormone biosynthesis, signaling transduction, receptor perception, and metabolism regulation. To verify the result of comparative transcription analysis, the expression profiles of DEGs were measured by qRT-PCR (Figure 6). The homolog gene of putative *EjIAA2* exhibited an increase tendency after 60 PAD and reached a peak on day 110. Similarly, the transcription increases of *EjIAA9* and *EjIAA14* were detected along with fruit development. *EjARF1* and *EjARF2* belonging to the homology genes of auxin response factor (ARF) demonstrated a transcript peak on days 130 and 140, respectively. An obvious increase of *EjARF3* was observed at the first 10-day interval (60−70 PAD), and sharply declined until 100 PAD. A continuous decrease of *EjARF3* was observed by the end of fruit ripening, except for a transient increase on day 110. Auxin efflux carrier components from the PIN-formed family, *EjPIN1* and *EjPIN3*, showed an opposite transcript profile. The transcript-level increase of *EjPIN1* began after 60 PAD and reached a maximum on day 130. *EjPIN3* was expressed mainly in the unripe fruit. Similarly, *F-box 2* (*EjAFB2*), an auxin response gene, also demonstrated a decreased tendency during fruit development.

**Figure 7:**
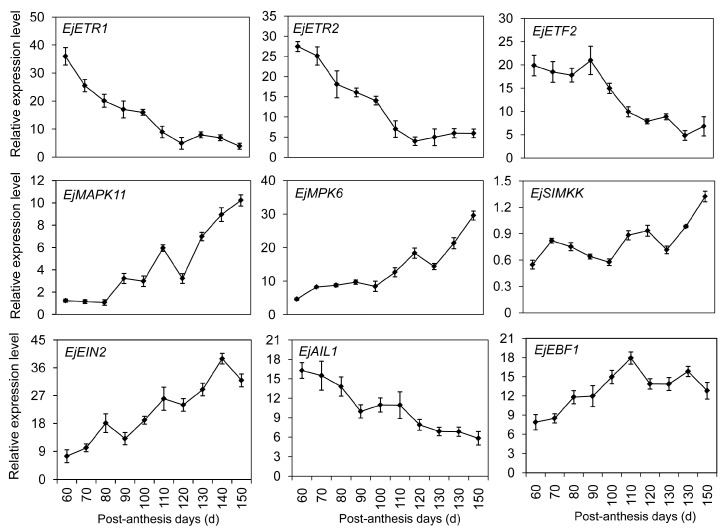
Transcription profiles of differentially expressed genes DEGs involved in the ethylene signal pathway using qRT-PCR. Each point is the mean of three determinations. Vertical bars, if larger than symbols, represent the standard error of the mean (*n* = 3).

Ethylene plays critical roles in many developmental events of climacteric fruits [3,6]. Little is known about its role in the ripening of non-climacteric fruits, especially the downstream components. That is because non-climacteric fruits often do not require ethylene to complete maturation. In our library, five DEGs were clustered in the ethylene signal pathway, including two ethylene receptors (*EjETR1* and *EjETR2*), one ethylene-responsive transcription factor (*EjETF2*), one APETALA2 (AP2)-like ethylene-responsive transcription factor (*EjAIL1*), and one ein3-binding F-box protein (*EjEBF1*). qRT-PCR results revealed that *EjETR1* and *EjETR2* had similar expression patterns (Figure 7). In young fruitlet, high transcription levels of *ETRs* were detected, and then sharply decreased from 110 to 150 PAD. No obvious difference was observed in the transcription levels of *EjETF2* from 60 to 90 PAD. Afterwards, a significant decrease was detected until ripening. Homology alignment displayed that some homologous genes (*EjMAPK11*, *EjMAPK6*, *EjSIMKK* and *EjEIN2*) to *Arabidopsis* involved in the ethylene signal pathway had been also identified in loquat fruit. Gradual increases of *EjMAPK11*, *EjMAPK6*, *EjSIMKK* and *EjEIN2* were detected during fruit development and ripening. *EjAIL1* exhibited a high transcription level at the initial stage, following a decline during the whole development stage. The opposite expression profile was observed in *EjEBF1*, and two peaks were observed on days 110 and 130.

### 2.8. Expansin Genes Related to Fruit Development

**Figure 8:**
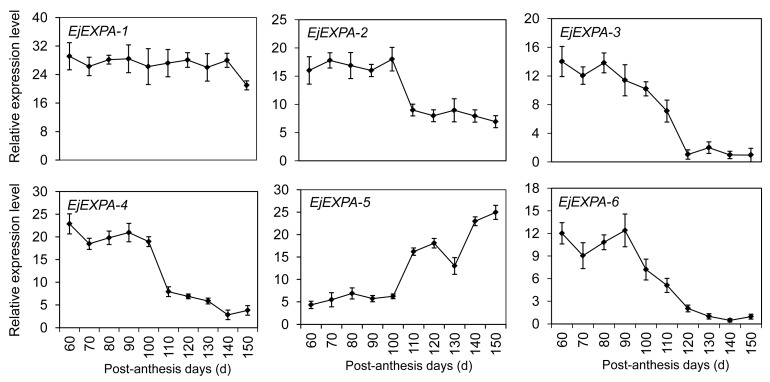
Expression profiles of differentially expressed genes DEGs involved in cell wall loosening and enlargement using qRT-PCR. Each point is the mean of three determinations. Vertical bars, if larger than symbols, represent the standard error of the mean (*n* = 3).

Cell wall loosening and enlargement play an important role in mediating fruit development and ripening. After BlastX analysis of sequencing data, six potentially distinct expansin genes (named *EjEXPA1–EjEXPA6*) were identified and clustered within the α-expansin family (Figure S1). The transcription analyses indicated that *EjEXPA2* and *EjEXPA4* were most abundant in young fruitlet, and then declined sharply to undetectable levels on day 110 (Figure 8). *EjEXPA5* was mainly expressed at the later stage of fruit development from 110 to 150 PAD. By contrast, *EjEXPA3* and *EjEXPA6* showed a down-up-down expression pattern with the maximum accumulation on day 80 and 90, respectively. However, no marked difference was detected in the transcription levels of *EjEXPA1* at all stages of fruit development except for 150 PAD.

### 2.9. Transcription Factors Involved in Fruit Development and Ripening

**Figure 9:**
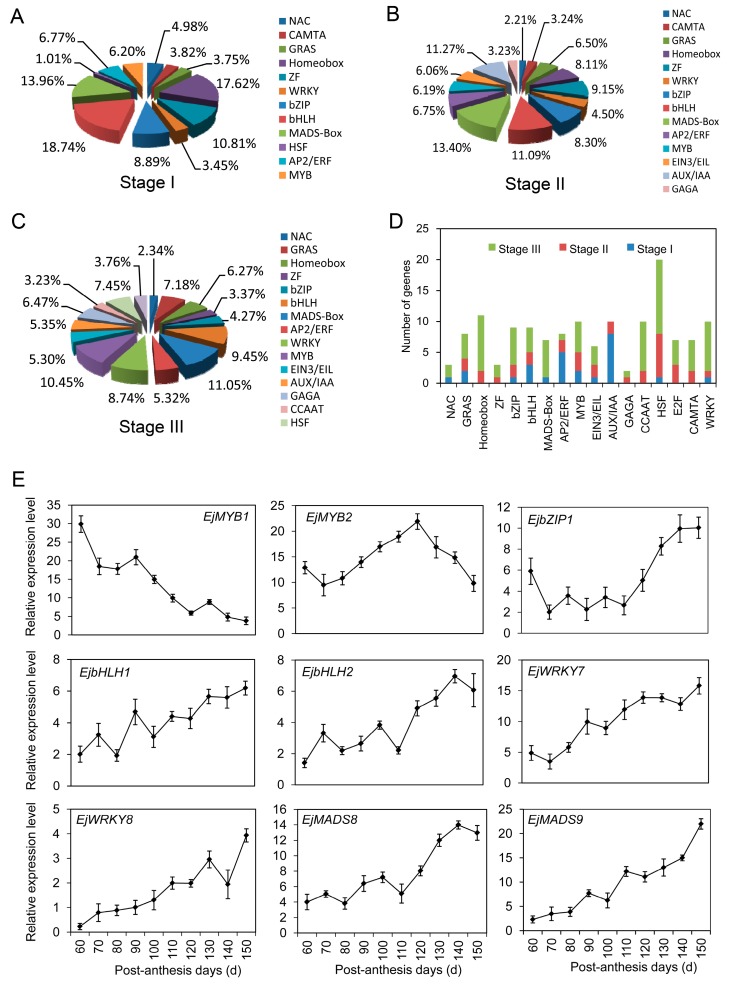
Distributions and expression profiles of transcription factors (TFs) during loquat fruit development. (**A**) Distributions and types of TFs at stage I; (**B**) Distributions and types of TFs at stage II; (**C**) Distributions and types of TFs at stage III; (**D**) Distributions and types of the differentially expressed TFs at three stages; (**E**) Expression profiles of the randomly selected TFs investigated by qRT-PCR. Each point is the mean of three determinations. Vertical bars represent the standard error of the mean (*n* = 3). bHLH, basic helix-loop-helix; bZIP, leucine zipper, ZF, zinc finger; HSF, hot shock factor; NAC, cup-shaped cotyledon; AP2/ERF, APETALA2/ethylene responsive factor; AUX/IAA, Auxin/indole-3-acetic acid; EIN3/EIL, ethylene-insensitive 3 and EIN3-like.

Specific TFs were necessary for controlling downstream gene transcripts. In our study, 195 TFs were identified at stage I, mainly including Aux/IAA, basic helix-loop-helix (bHLH), homeobox, basic leucine zipper (bZIP), zinc finger (ZF), and MADS-box (Figure 9A). Within stage II, 204 TFs contained bHLH, MADS-box, ethylene-insensitive 3 and EIN3-like (EIN3/EIL), MYB, APETALA2/ethylene responsive factor (AP2/ERF), Auxin/indole-3-acetic acid (AUX/IAA), homeobox, ZF, and bZIP (Figure 9B). Among 236 TFs involved in stage III, MADS-box, bHLH, MYB, WRKY, hot shock factor (HSF), cup-shaped cotyledon (NAC), GRAS, and homeobox proteins were abundantly presented (Figure 9C). Overall, 136 unigenes putatively encoding TFs of diverse families were differentially expressed at different development stages of loquat fruit. The well-represented categories mainly included HSF, homeobox, ZF, bHLH, bZIP, WRKY, GRAS, GAGA-binding, EIN3/EIL, and CCAAT-binding proteins (Figure 9D; Table S3). Combining with the enrichment analyses of TFs and sequence elements, TF binding to the target sequences to regulate gene transcription will be investigated in our future research.

The expression profiles of nine TFs were determined by qRT-PCR. *EjMYB1* presented a decrease tendency during fruit development, while a down–up–down expression pattern was found in *EjMYB2*. *EjbZIP1* had a high transcript level in young fruitlet, and markedly decreased on day 70. After the stable expression level was kept from 70 to 110 PAD, an obvious increase was detected until fruit ripening. Transcripts of *EjbHLH1* and *EjbHLH2* demonstrated the irregular increases from 60 to 150 PAD. *EjWRKY7* and *EjWRKY8* increased 3-fold and 4-fold in comparison with the initial values, respectively. Similarly, transcripts of *EjMADS8* and *EjMADS9* were dramatically accumulated during fruit development regimes, and particularly at the later stage of fruit ripening.

## 3. Discussion

The development and ripening of fruits have received considerable scientific scrutiny because of both the uniqueness of these biological processes and their importance for the human diet. Despite their varied morphologies, fruits share common events and pathways in their life cycle. Physicochemical measurements, especially TSS, TA and sugar accumulation, are considered as current standards for objectively evaluating fruit quality [25,26]. In our study, continuous and progressive increases of TSS and TA were observed during loquat fruit development and the highest contents were recorded on day 150. The increase of TA was attributed to the biosynthesis of organic acids [1,8]. A rise of TSS caused the depolymerization of polysaccharides and conversion of fruit starch to sugars [27]. A significant increase was also detected in the TSS/TA ratio from 90 to 150 PAD (Figure 1D). Our results supported that the phase of rapid fruit growth was accompanied by sugar accumulation due to an increase in the phloem translocation rate [26]. In ripe fruit, sucrose, glucose and fructose were present in cv. Jiefangzhong loquat flesh, while fructose was the most dominant sugar. The soluble sugar distributions mainly depend on fruit cultivars and development stages [28,29,30].

To elucidate the molecular mechanism of fruit development, loquat fruit samples from three different stages were successfully sequenced using the Illumina technique. Our data provided an overview that DEG transcription characteristics and signal networks were involved in fruit development. In the carbohydrate signal pathway, most of the sugar metabolism genes like *EjSS* and *EjSPS* were identified. High expression levels of *EjSS-S* implied that sucrose accumulation was a dominated process at stage I (Figure 5). However, *EjSS* was mainly involved in sucrose cleavage rather than sucrose synthesis from 100 to 150 PAD. The increases in the expression levels of *EjSPS1* and *EjSPS2* uncovered that soluble sugars were produced via synthesis of sucrose, followed by hydrolysis into glucose and fructose. The similar pattern of sugar accumulation was also shown in kiwifruit and citrus [31,32]. Once sucrose was converted to fructose and glucose by invertase or SS-C in sink cells, the regulation proteins were immediately phosphorylated by hexokinase and fructokinase [31]. To complete the metabolism reaction, the tricarboxylic acid (TCA) cycle provides energy (ATP), reducing power [NAD(P)H] and precursors for the synthesis of many secondary metabolites [33]. The abundant unigenes related to carbohydrate metabolism and TCA cycle were also presented in Table 2 and Figure 4C. For example, 14-3-3 proteins can directly or indirectly interact with SPS to mediate sucrose synthesis. The interaction protected SPS from the attack of proteinase [34].

Consistent with sugar metabolism, most DEGs were involved in plant hormone signal pathways (Figure 4C). *EjAux-IAA* and *EjARF* genes showed dramatic alterations in their expression levels during fruit development (Figure 6). Aux/IAA had been shown as transcriptional repressors through interactions with ARF proteins that directly bind to auxin-responsive elements in the promoters of auxin responsive genes [35]. The reduction of *IAA9* transcription levels in tomato resulted in fruit development prior to pollination and fertilization by the formation of ARF-Aux/IAA heterodimers [36]. Like *IAA9*, for other auxin response genes, such as *IAA2* and *IAA14*, the transcription levels were also induced by auxin treatment [37]. We found that *EjARF3* transcript accumulation happened in cell division and early fruit development (Figure 6). The *ARF3* silencing caused a dramatic increase of chlorophyll content resulting in a dark green unripe fruit [38]. Additionally, the members of the PIN family were also identified during loquat fruit development. Although the roles of PINs have been extensively studied in vegetative development, their functions are less clear in non-climacteric fruit development [35]. qRT-PCR results indicated that high expression levels of *EjPIN3* were detected at the initial stage of fruit development. A similar expression pattern of *PINs* were exhibited in immature tomato fruit, coinciding with a high rate of auxin transport via the pedicel, which likely prevents premature abscission during fruit development [39]. Interestingly, the transcription pattern of *EjPIN1* appeared to be consistent with an active IAA-related metabolism during fruit ripening (Figure 6), significantly upregulated by ethylene rather than by auxin [40]. This finding indicated that a cross-talk between auxin and ethylene might occur in the regulation of loquat fruit development.

Ethylene production in climacteric and non-climacteric fruits has been reported during different development stages [3,13]. Some regulation elements of ethylene biosynthesis and perception were also found in our transcriptome data. High transcription levels of *EjETR-1*, *-2* and *-3* suppressed the expressions of ethylene-responsive genes in young fruitlet (Figure 7). However, three *EjETRs* of strawberry showed much higher expression levels in ripening fruit [41]. This may be related to other functions of ethylene such as starch/sugar metabolism, fruit coloring, or aroma volatile synthesis [42]. For example, exogenous ethylene application stimulated the pigment changes of chlorophyll degradation and carotenoid biosynthesis in citrus [43], and enhanced anthocyanin accumulation in grape berries [44]. Interestingly, *EjERF2* transcript was detected with considerably high levels from 60 to 90 PAD, but fruit size was hardly increased with low auxin content and ethylene biosynthesis. Considering the high contents of endogenous H_2_O_2_ production during the early stage of fruit development, the transcription increase of *EjERF2* could be altered due to H_2_O_2_ accumulation. Similarly, exogenous application of H_2_O_2_ could upregulate the *ERF* expression levels during plum fruit development [45]. These results supported the intriguing hypothesis that ethylene-dependent and -independent common genes in both types of fruits were possibly regulated by the conserved and primary regulators through evolution [27].

Our qRT-PCR results exhibited that expansin genes identified in RNA-seq data displayed different transcripts during fruit development and softening (Figure 8). Phylogenetic alignment of expansin proteins (EjEXP1-6) revealed the existence of at least two families (α- and β-expansins) (Figure S1). EjEXP1-6 were grouped into a similar α-family with a distinct and overlapping expression during fruit development. *EjEXP2* was selectively expressed during a period of rapid cell expansion, but not during fruit ripening (Figure 8). Interestingly, *EjEXP4* with a similar expression profile was aligned within a divergent phylogenetic subgroup, which might be related to its unknown biological function. In tomato, *LeEXP4* was detected specifically in flowers and expanding fruits [46]. The stable expression levels of *EjEXP1* were maintained during the whole fruit development, which suggested that the basic action mechanism of *EjEXP1* is likely to be similar in both expanding and ripening fruit. *EjEXP1* was also involved in chilling-induced lignifications of the postharvest loquat fruit [15]. Therefore, the divergent expansins in specific cell types were mediated differentially by environmental and hormonal stimuli. More detailed analyses of tissue-specific expression and hormonal response of each expansin may help elucidate the evolutionary and functional divergence.

TFs are necessary for controlling various cellular processes through the regulation of gene expression. A total of 136 diverse TFs had been identified as pivotal regulators during loquat fruit development. One of the most abundant TFs, MADS proteins, were not only identified in tomato, but also in non-climacteric fruits [47]. A ripening-inhibitor (*RIN*) gene of tomato, belonging to the MADS family, activates a series of downstream ripening genes by forming a heterotetrameric complex with FUL/TDR4 FUL2 and/or TAGL1 [48]. A RIN homology in loquat was also identified during fruit development. Suppression of a *SEPALLATA1/2*-like gene either in apple (*MdMADS8/9*) or in strawberry (*FaMADS9*) led to a great decrease in fruit flesh [47]. The observed results imply that at least some aspects of fruit development and ripening are shared between climacteric and non-climacteric fruits. Notably, WRKY and HSF, involved in plant response to biotic and abiotic stresses, were also observed in loquat fruit. The accumulated evidence indicated that the WRKY family also participated in regulating fruit maturation and hormone signaling [49]. Under abscisic acid stimulation, WRKY40 relieves the inhibition for ABI5 by formatting the complex with ABAR [49,50]. The high transcription levels of *EjHSF1* and *EjHSF2* at stage III supported the prediction that *HSFs* might be associated with regulating fruit ripening. The recent evidence indicated that *HSFs* also participated in the response regulation of low temperature-induced lignifications in loquat fruit [51]. Moreover, MYB1 and MYB2 can bind directly the promoters of *PAL1* or *CL1/5* to modulate lignin biosynthesis [14]. A suppression complex between MYB and AP2/ERF showed a negative correlation with lignifications [16]. Some biochemical data also supported that MYB in complexes with bHLH and WD40 proteins could regulate anthocyanin and flavonoid accumulation in the fleshy fruit [52,53]. However, further research is necessary to ascertain whether bHLH and MYB cooperate with other proteins to regulate loquat fruit development and ripening. Additionally, other TFs, including bZIP, NACs, GRAS, homeobox and ZF, had also been identified in our transcriptome data of loquat fruit. The expression levels of *bZIP1* were induced by wounding in tomato leaf [54], and improved by C_2_H_4_ in tobacco leaf [55]. *HY5* and *RF2a* (bZIP family) were also detected in the mature-fruit abscission zone of olive [56] and melon [57]. *NACs* manifested the increase of transcripts in mature or immature fruit abscission zones [58,59]. Therefore, these TFs have been implicated in diverse processes, including a developmental program, senescence, stress response, and hormone signaling [45,53,55,60,61], but their functional characteristics and molecular mechanism still need to be elucidated. The RNA-seq data will help obtain better information about gene transcription and metabolism networks, including those that previously had not been considered to be differentially expressed during fruit development and ripening.

## 4. Materials and Methods

### 4.1. Plant Materials

Field-grown loquat fruit (*Eriobotrya japonica* Lindl., cv. Jiefangzhong) was used in this study. All samples were collected from 12-year-old trees grown in the Loquat Germplasm preservation orchard of South China Agricultural University, Guangzhou, China. Loquat fruits with yellow pericarp were harvested in May 2015. The uniform fruit was selected for size, weight and color without signs of disease and pest damage. Fruit firmness was about 4.0 N and TSS content was about 11.0% based on our previous study [28]. Twenty-five fruits from three trees were randomly selected at 10-day intervals from 60 to 150 PAD. The sliced tissues from three independent fruits were pooled together as one of three replications at each sampling period. All samples were immediately frozen in liquid nitrogen, and stored at −80 °C before being analyzed.

### 4.2. Determination of Sugar Content

Sugar content was measured according to the description of Song et al. [28]. Five grams of flesh tissues were homogenized in 20 mL of pre-cold ethanol (95%, *v*/*v*). The extract was filtered through a 0.2 μm filter, and 6 mL of the extract was passed through a Sep-Pak C18 cartridge (Waters, Milford, MA, USA) activated with acetonitrile and water. A 20 μL aliquot was injected into a high-performance liquid chromatography system in a Waters chromatograph equipped with a refractive index detector and a CarboSep CHO-682 carbohydrate analysis column (Transgenomic, San Jose, CA, USA). Individual sugar was identified and quantified by comparison with retention times and peak areas of individual sugar standard. The content was expressed as g·kg^−1^.

### 4.3. Measurement of TSS and TA

Five grams of flesh tissue from each replicate were homogenized and then centrifuged at 13,000× *g* for 20 min. The supernatant was used to analyze TSS and TA contents [10]. TSS (%) was determined using a digital pocket refractometer (PAL-a, ATAGO, Tokyo, Japan). TA was measured by titration with NaOH (0.1 mol·L^−1^) to pH 8.1 and expressed as % malic acid (mass/mass).

### 4.4. RNA Isolation and cDNA Synthesis

Total RNA was isolated from loquat fruit using Trizol reagent (Life Technologies Inc., Rockville, MD, USA). The SMART™ PCR cDNA Synthesis Kit (BD Biosciences Clontech, CA, USA) was used for cDNA synthesis from 1 μg of poly (A^+^) RNA according to the manufacture instruction. An equal amount of total RNA from each fruit development time was pooled for library preparations. The first-strand cDNA was amplified with PCR primers provided in the SMART™ PCR cDNA Synthesis Kit. The PCR mixture (50 μL) contained 1× Advantage 2 PCR reaction buffer, 0.3 mmol·L^−1^ primers, 1× Advantage 2 Polymerize mix, 200 mmol·L^−1^ dNTPs and 5 ng first-strand cDNA. Sixteen PCR cycles (95 °C for 7 s, 65 °C for 20 s, and 72 °C for 3 min) were performed. The products were used for DSN normalization and cDNA library construction.

### 4.5. First-Strand cDNA Normalization and Single-Strand DNA Amplification

After purification using the Qia Quick PCR Purification Kit (Qiagen, Tokyo, Japan), PCR products were dissolved in milliQ water to 100 mg·L^−1^ of cDNA concentration. The first-strand cDNA was normalized according to the description of Zhulidov et al. [62]. The reaction was subsequently terminated by adding 10 μL of 5 mmol·L^−1^ ethylenediaminetetraacetic acid (EDTA).

The normalized single-strand DNA (ssDNA) was amplified in 50 μL of reaction mixture using the Advantage 2 PCR kit (BD Biosciences Clontech). The reaction mixture contained 1× Advantage 2 Polymerize mix, 0.3 mmol·L^−1^ CapM primers (Table S1), 200 mmol·L^−1^ dNTPs, and 1× Advantage 2 PCR reaction buffer. Twenty-five PCR cycles (95 °C for 7 s, 65 °C for 20 s, and 72 °C for 3 min) were performed to obtain a concentration of ca. 20 mg·L^−1^ ssDNA samples.

### 4.6. cDNA Library Construction and Illumina Sequencing

After normalization, ssDNA samples were digested by *Sfi*I and were cloned into the pDNR-LIB vector (Promega Corp., Madison, WI, USA). Frequencies of the corresponding cDNA sequences in the library were calculated from the number of positive colonies. For analysis of the insert size distribution, colonies picked randomly from the fruit library were identified using PCR with standard M13 primers (Table S4). The reaction system was performed by denaturation at 94 °C for 10 min, 30 cycles (94 °C for 30 s, 56 °C for 30 s, and 72 °C for 2 min), and 72 °C for 10 min. The PCR products were detected on 1.2% (*w*/*v*) agarose gel with a 1.0 kb molecule marker (Takara, Dalian, China). The cDNA libraries were sequenced.

### 4.7. Sequence Processing and Annotation

Image deconvolution and *Q*-value were calculated in the Illumina data processing pipeline (version 1.6, Chicago, IL, USA). Before assembly, adaptor sequences, empty reads and low-quality sequences were removed. Data evaluation was performed according to the description of Müller-Herbst et al. [63]. To annotate isotigs and singletons, the non-redundant sequences were searched against the protein databases obtained from NCBI with a search threshold of *E*-value cut-off 10^−5^ [18]. Based on the BlastX top hit genes [24], the abundant sequences were used to obtain further information on the function and motif through the InterPro member databases. The full-length cDNAs at 5′ end were analyzed from BlastX output. Functional annotation by GO terms was carried out based on the best hits from nr annotation using Blast2GO [23]. Annotation of KEGG pathways was performed by sequence comparisons using a BlastX algorithm against the KEGG database with an *E*-value threshold of 10^−5^ [64]. The results were summarized according to their molecular functions, biological processes and cellular components.

### 4.8. Gene Expression Analysis by qRT-PCR

qRT-PCR was performed using the first-strand cDNA as templates on a LightCycler^®^ 480 Real-Time PCR system (Roche Diagnostics, Vienna, Austria), with 10 µL FastStart Universal SYBR Green I Master (ROX, Roche Diagnostics, Mannheim, Germany), 1 µL forward primer (10 µmol·L^−1^), 1 µL reverse primer (10 µmol·L^−1^), 1 µL cDNA (10 ng) and 7 µL water. The LightCycler experimental cycle was as followed: denaturation program (95 °C for 5 min), amplification and quantification program repeated 40 cycles (95 °C for 10 s, 60 °C for 10 s, 72 °C for 70 s), melting curve program (60–95 °C with a heating rate of 0.1 °C per second and a continuous fluorescence measurement). Expression of the *β-actin* gene was used as an internal control to normalize the amount of mRNA. The primer sequences are shown in Table S4.

### 4.9. Statistical Analysis

Twenty-five fruit samples were randomly selected at each time point of fruit development. All data were expressed as mean ± standard error (SE) and subjected to statistical analysis with SPSS version 13.0 (SPSS Inc., Chicago, IL, USA). One-way analysis of variance (ANOVA) was performed to determine the effects of sampling time point on sugar accumulation, TA, TSS and DEG expressions. The data represent the mean values obtained from three independent biological replicates. Significant differences between means were determined by Tukey post hoc comparison tests at *p* < 0.05.

## 5. Conclusions

This study investigated the transcriptome profiles of loquat fruit during development and ripening using Illumina RNA-seq and DEG deep-sequencing technologies. A total of 48,838 unigenes were assembled and 31,034 unigenes were annotated using GO terms. Genes related to fruit quality and fruit softening were found, and their expression profiles were detected throughout ten fruit development times. Several hormone metabolism genes and TFs involved in fruit development were also identified using transcriptome analysis and qRT-PCR. These findings provide a platform for further functional genomic research on this fruit crop and a reference for studying complicated metabolic processes in non-model perennial species. The DEGs will be certainly valuable for elucidation of molecular mechanisms in fruit development.

## Figures and Tables

**Table 1:** Summary of sequence assembly.

Assembly Metric	Sequences (No.)	Base Pairs (bp)	Mean Length (bp)
Raw sequencing reads	51,881,623	5,019,322,712	97
Clean reads	51,610,234	4,850,271,306	94
Contigs (≥100 bp)	453,421	125,597,617	277
Singletons (≥150 bp)	32,608	20,575,648	631
Clusters (≥150 bp)	16,230	18,047,760	1112
Total unigenes (≥150 bp)	48,838	38,623,512	790

**Table 2:** List of 30 top hit DEGs from RNA-Seq.

Unigene	Putative Function	Accession No.	Source Organism	*E*-Value ^a^
Contig 5600	Polyphenol oxidase	AEY79824	*Triticum aestivum*	5.17 × 10^−9^
Contig 5601	Pyrophosphate-fructose 6-phosphate	XP008453023	*Cucumis melo*	3.11 × 10^−7^
Contig 5602	Ethylene cinnamyl alcohol dehydrogenase	XP017179614	*Malus domestica*	5.84 × 10^−7^
Contig 5603	ADP-glucose pyrophosphorylase subunit	ABK97520	*Sorghum bicolor*	3.52 × 10^−5^
Contig 5604	Sucrose phosphate synthase	AAL86360	*Actinidia chinensis*	3.24 × 10^−9^
Contig 5605	Heat shock protein 70	NP567510	*Arabidopsis thaliana*	2.25 × 10^−6^
Contig 5606	Ethylene response factor	JF815559	*E. japonica*	0.0
Contig 5607	Expansin	EU123922	*E. japonica*	0.0
Contig 5608	TFL1-like protein	BAD10969	*Pyrus communis*	7.13 × 10^−5^
Contig 5609	Ethylene-responsive transcription factor	JF815559	*E. japonica*	1.84 × 10^−11^
Contig 5610	Ethylene receptor	NP_00128083	*M. domestica*	1.56 × 10^−7^
Contig 5611	Phenylalanine ammonia lyase	EF685344	*E. japonica*	0.0
Contig 5612	ATP synthase subunit	NP568203	*A. thaliana*	1.28 × 10^−7^
Contig 5613	Fructokinase	JF414124	*E. japonica*	0.0
Contig 5614	Hexokinase	JF414121	*E. japonica*	0.0
Contig 5615	Catalase 1	XP008348924	*M. domestica*	0.0
Contig 5616	Ribulose-1,5-bisphosphate carboxylase	XM003616402	*Medicago truncatula*	2.84 × 10^−5^
Contig 5617	Sucrose synthase	XM002266984	*Vitis vinifera*	0.0
Contig 5618	Phosphoglycerate kinase	M008384936	*M. domestica*	2.03 × 10^−7^
Contig 5619	Lipid transfer protein 4 precursor	AY793558	*Lens culinaris*	5.79 × 10^−7^
Contig 5620	Myb transcription factor	XM008366832	*M. domestica*	1.23 × 10^−5^
Contig 5621	UDP-glucosyltransferase	DR993941	*M. domestica*	1.10 × 10^−9^
Contig 5622	Ethylene response factor (ERF)	JF412350	*M. domestica*	2.22 × 10^−8^
Contig 5623	Senescence-related protein	XM002300415	*Populus trichocarpa*	2.05 × 10^−6^
Contig 5624	Poly(A)-binding protein	NM001198255	*A. thaliana*	0.0
Contig 5625	Metallothionein-like protein	AF009959	*M. domestica*	5.89 × 10^−7^
Contig 5626	Translation elongation factor	XM008340889	*M. domestica*	1.85 × 10^−8^
Contig 5627	DELLA protein	KC434135	*Triticum aestivum*	1.11 × 10^−8^
Contig 5628	Vacuolar Ca^2+^/H^+^ exchanger	AB012932	*Vigna radiata*	2.09 × 10^−10^
Contig 5629	ATP binding protein	XM002521829	*Ricinus communis*	0.0
Contig 5630	Phosphoric diester hydrolase	NM101237	*A. thaliana*	1.66 × 10^−5^
Contig 5631	Serine/threonine-protein kinase	XM008391178	*M. domestica*	2.42 × 10^−5^
Contig 5632	Cytochrome C reductase	X79275	*Solanum tuberosum*	2.03 × 10^−7^
Contig 5633	1-Aminocyclopropane-1-carboxylic acid oxidase	AB003514	*Actinidia deliciosa*	1.24 × 10^−8^
Contig 5634	Vacuolar ATP synthase subunit G1	NM180158	*A. thaliana*	1.34 × 10^−7^
Contig 5635	β-1,3-Glucanase	AY548364	*M. domestica*	2.24 × 10^−8^
Contig 5636	Alcohol dehydrogenase	L23548	*Zea mays*	2.54 × 10^−6^
Contig 5637	14-3-3 Family protein	NM001247178	*S. lycopersicum*	2.45 × 10^−5^
Contig 5638	MADS box genes	AJ000759	*M. domestica*	0.0

^a^ *E*-value obtained by BlastX analysis [24].

## Supplementary Materials

Supplementary materials can be found at www.mdpi.com/1422-0067/17/11/1837/s1.
