# Supplementary material for: Comparative Transcriptional Analysis of Loquat Fruit Identifies Major Signal Networks Involved in Fruit Development and Ripening Process
Source: Int J Mol Sci. 2016 Nov 4;17(11):1837. doi: 10.3390/ijms17111837 (PMC5133838; doi:10.3390/ijms17111837)
Supplement: Supplementary file 1 [file ijms-17-01837-s001.zip › ijms-143776-Supplementary Materials/ijms-143776-supplementary.pdf]

Supplementary Materials: Comparative Transcriptional Analysis of Loquat Fruit Identifies Major Signal Networks Involved in Fruit Development and Ripening Process

Huwei Song, Xiangxiang Zhao, Weicheng Hu, Xinfeng Wang, Ting Shen and Liming Yang

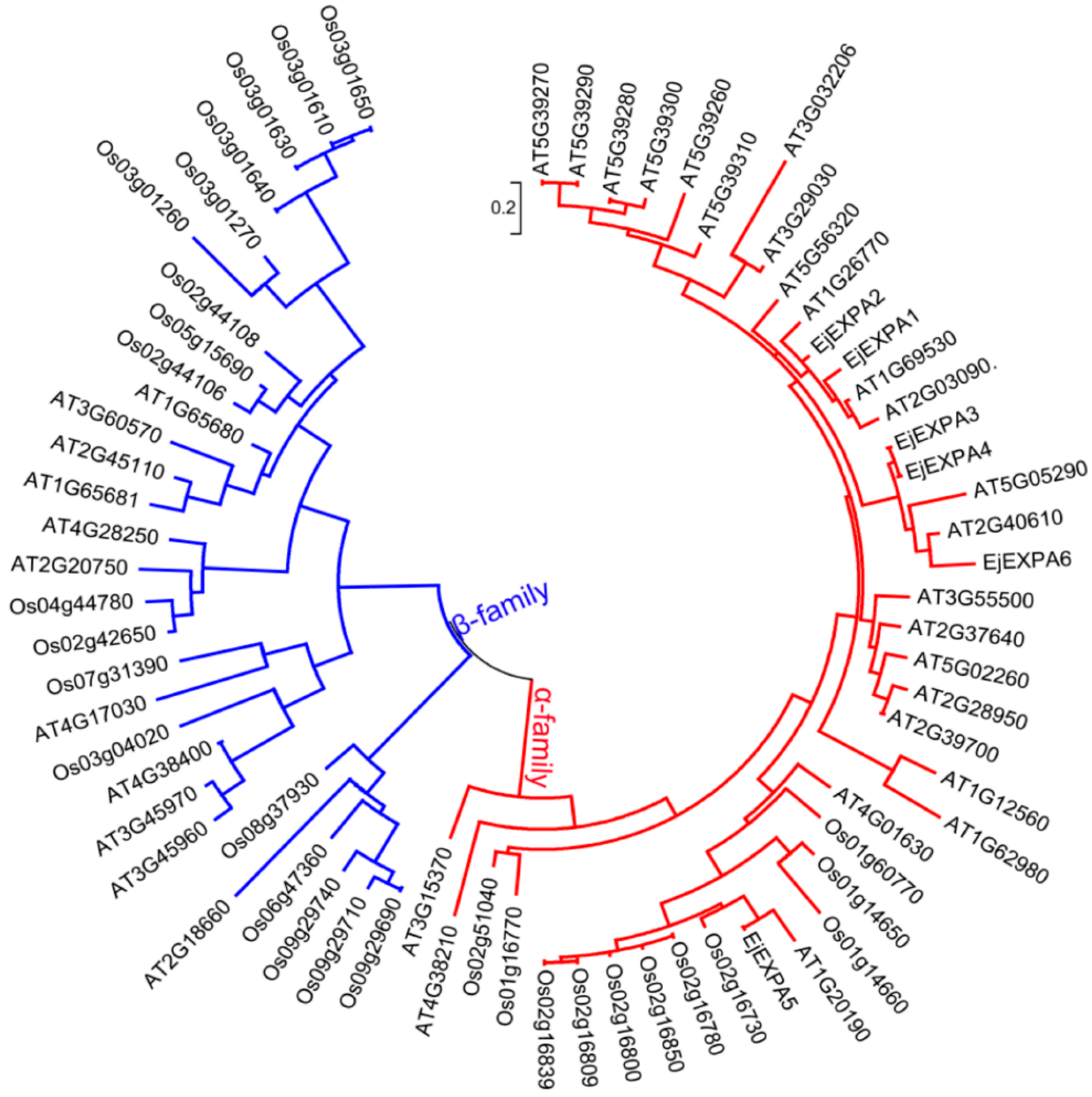

Figure S1. Phylogenetical tree of the deduced EjEXPA1-6 protein sequences in loquat fruit.

Table S1. Overview reads of the abundance categories among the three libraries.

Sample ID	ReadSum	BaseSum	GC (%)	Q20%	Q30%
E. japonica-S1	31864876	3134723235	48.72	98.62	94.78
E. japonica-S2	31947603	3421678905	47.89	98.53	94.61
E. japonica-S3	38939218	3935124956	48.63	98.64	94.85

Table S4. Primer sequences used in this study.

Primer Name		Primer Sequence (5′ to 3′)
SMART oligo IV		AAGCAGTGGTATCAACGCAGAGTGGCCATTACGGCCrGrGrG
CDS-3M adapter		AAGCAGTGGTATCAACGCAGAGTGGCCGAGGCGGCC(T)20 KN
M13	Forward	TGTAAAACGACGGCCAGT
	Reverse	CAGGAAACAGCTATGACC
EjEXPA1	Forward	ATCTTTGGGAACCCTAACTC
	Reverse	CTCGGCTGATTTACAACAAT
EjEXPA2	Forward	GCTGCGGGGCTTGTTTTGAG
	Reverse	CGGCCGTCGCTGGTTGTGA
EjEXPA3	Forward	CCCGGCAGCATCATCGTC
	Reverse	CTGCCGTCGCTGGTGGTG
EjEXPA4	Forward	CTGCGGGTCTTGCTATGAG
	Reverse	GGCCGTTGAGGTAAGAGTT
EjEXPA5	Forward	CTCCCTGGCTCCATCATAGTC
	Reverse	GGGCAACATTGTAAGAAACC
EjEXPA6	Forward	AACCCGCCTCGCACCCACTT
	Reverse	ACGCCCTTTACAGCCACCTCAG
EjSPS1	Forward	GTTGTCCGCATCCCCTGTGG
	Reverse	GATCTCTTGCCTGGTGCTGGT
EjSPS2	Forward	GATACTGGTGGTCAGGTTAAG
	Reverse	GTCTGCGTAATGCCATGGATG
EjSS-C	Forward	AGGCGGCACTGATTGTAACG
	Reverse	CTTGCTTCGGGTATGAGTCTT
EjSS-S	Forward	GCGCACGCATTGGAGAAAAC
	Reverse	CGATCGCTTAGGGTGCCAAT
EjMAPK11	Forward	GGACACGATCGTAGCTCA
	Reverse	GATCCTGATCGGTAGCTAGC
EjEIN2	Forward	CCTGCGGCTCGATCTTCCAT
	Reverse	GCAATCTCGCCTCTCATCCA
EjSIMKK	Forward	GTACACCGGATGCCCATACT
	Reverse	AGGGCTCATGCGGATCGTC
EjMAPK6	Forward	CATTGGCGCTGACTAGGTA
	Reverse	GCCAGTCCTTGATCCTACC
EjIAA2	Forward	GTTAAAATTAACAACAAGC
	Reverse	GAAGCCAATTTGAAACTCA
EjIAA9	Forward	GATCTGGATTTGTACCAAC
	Reverse	GGATCCCTTCATGATTCTGA
EjIAA14	Forward	CGAGTCATGCAAACGTTTGC
	Reverse	CATGATCTGTTCTTGAACTT
EjARF1	Forward	ATGCTGTTCTCTTGGTGTTTG
	Reverse	GAGAGTGCAGCCCCAGCTT
EjARF2	Forward	CTCTCCAAGGGCGATGCAAT
	Reverse	CAAAGCACGTGGACGTATT
EjARF3	Forward	GACTTAAAGCAGCCACTGT
	Reverse	GCATCCAGGCCCACCATCA
EjPIN1	Forward	CTACCACTAGAGAAGCCG
	Reverse	GTCAGAAACACGAGTCGC
EjPIN3	Forward	GAACATGACTCCACGGCCG
	Reverse	CGACCACCGGGGAAACCCA

Table S4. *Cont.*

Primer Name		Primer Sequence (5′ to 3′)
EjAFB2	Forward	GAGATACAGACAAAGGCAT
	Reverse	CATCAGCAAGAAGCGCCGT
EjETR1	Forward	GACGGGTAGGCATGTGAGA
	Reverse	GCATAGCGTTTTGTTGAGAG
EjETR2	Forward	TTGCGCTTGCTTATTTCTCCA
	Reverse	GGCATCCACAAGGCACACTC
EjETF2	Forward	AAGCCCAAACCATCTATCAC
	Reverse	CACCCGCCACCTCATTCTTA
EjAIL1	Forward	CATTCGCAACTGAAGAGG
	Reverse	GCTGCTCCGCCAACTGGAAG
EjEBF1	Forward	CTGAGCCACTGTCGTTCAA
	Reverse	GTTTCCAATATTTGGACAG
EjMYB1	Forward	CCCAAGTCATGGCCCTTTG
	Reverse	GCAACCGTGACCACAGCGT
EjMYB2	Forward	GAGCTGCAGGCTGCGGTGGA
	Reverse	GTTTCCGAGGAGGCTATGGA
EjZIP1	Forward	GCGCTGCTGCAGGAGTTCA
	Reverse	GAGCGCTCGAGGAACTCGA
EjbHLH1	Forward	GCCTTGTCGCCGGAGGATT
	Reverse	GTGATGTTTCTTTGAATAAGC
EjbHLH2	Forward	GATGGCTCTAATAATATGGAT
	Reverse	CCCAACTAATTGCAGTCTCA
EjWRKY7	Forward	ATCAGAGCTCTTACATGGC
	Reverse	GACAGCCCACGGCGCCGCC
EjWRKY8	Forward	GCAGAAGCAGCTCAAGGACG
	Reverse	GATCTCCTTGATGCAGCCGTC
EjMADS8	Forward	CTCTATGAATTTTCCAGTTCC
	Reverse	CTGCATGTTATGTTCGGTAG
EjMADS9	Forward	ATGCACGAGTACTGTAGCCCT
	Reverse	CAGAGCCTCTTCCCAGACTG
β-actin	Forward	TCAGGAGCAACACGAAGT
	Reverse	TTGGTATGGGTCAGAAGG
